# Molecular Detection of Microsporidia in Rabbits (*Oryctolagus cuniculus*) in Tenerife, Canary Islands, Spain

**DOI:** 10.3390/biology11121796

**Published:** 2022-12-10

**Authors:** Edgar Baz-González, Natalia Martin-Carrillo, Katherine García-Livia, Néstor Abreu-Acosta, Pilar Foronda

**Affiliations:** 1 Department Obstetricia y Ginecología, Pediatría, Medicina Preventiva y Salud Pública, Toxicología, Medicina Legal y Forense y Parasitología, Universidad de La Laguna, 38203 San Cristóbal de La Laguna, Spain; 2 Instituto Universitario de Enfermedades Tropicales y Salud Pública de Canarias, Universidad de La Laguna, 38203 San Cristóbal de La Laguna, Spain; 3 Nertalab S.L., 38008 Santa Cruz de Tenerife, Spain

**Keywords:** microsporidia, *Encephalitozoon cuniculi*, PCR, rabbit, *Oryctolagus cuniculus*, Canary Islands

## Abstract

**Simple Summary:**

Microsporidia are a group of fungal-related pathogens widely distributed in the environment, with some species having a negative impact on animals and public health. The European rabbit (*Oryctolagus cuniculus*) is considered the natural host of *Encephalitozoon cuniculi*, a microsporidian pathogen of mammals, including humans. The infection caused by *E. cuniculi*, encephalitozoonosis, ranges from asymptomatic to severe lesions in rabbits, with clinical signs involving the central nervous system, kidney, and eye being the most common. The majority of reported cases have been in domestic rabbits, while cases in wild rabbits are uncommon. Due to the lack of data on microsporidia in the Canary Islands, the aim of this work was to analyze the prevalence and identity of microsporidia in fecal samples from rabbits in Tenerife.

**Abstract:**

*Enterocytozoon bieneusi* and *Encephalitozoon* spp. are microsporidia with zoonotic potential that have been identified in humans, as well as in a large group of wild and domestic animals. Several wildlife species have been studied as reservoirs of zoonotic microsporidia in mainland Spain, including the European rabbit (*Oryctolagus cuniculus*). Due to a lack of data on microsporidia infection in wildlife on the Canary Islands, the aim of this work was to analyze the prevalence and identify the species of microsporidia in rabbits in Tenerife. Between 2015 and 2017, a total of 50 fecal samples were collected from rabbits in eight municipalities of Tenerife, Canary Islands, Spain. Seven of the fifty samples (14%) were amplified using nested polymerase chain reaction (PCR) targeting the partial sequence of the 16S rRNA gene, the internal transcribed spacer (ITS) region, and the partial sequence of the 5.8S rRNA gene. Sanger sequencing reveals the presence of *Encephalitozoon cuniculi* genotype I in two samples (4%), and undescribed microsporidia species in five samples (10%). This study constitutes the first molecular detection and genotyping of *E. cuniculi* in rabbits in Spain.

## 1. Introduction

The European rabbit (*Oryctolagus cuniculus*) is one of the most successful invasive mammals. Ancestors from its native Iberian range have been introduced to every continent except Antarctica and over 800 different islands or island groups, although with mixed success [1]. This lagomorph was introduced to the Canary Islands, a Spanish archipelago located in northwest Africa (13°23′–18°8′ W and 27°37′–29°24′ N), in around the 15th century. This species is distributed throughout the archipelago, and has become a highly sought-after hunting resource [2]. In 2017, rabbit abundance was estimated at a mean value of 2.22 individuals/ha in Tenerife, with a standard deviation of 2.25 individuals/ha, suggesting that there is high spatial variability in the abundance of the species. In general, abundance is higher in areas of low elevation and slope [3]. Population density is influenced by the presence of predators, hunting activity, and the incidence of infectious diseases such as myxomatosis, rabbit hemorrhagic disease, and coccidiosis [2].

A group of pathogens that affects rabbits worldwide is the phylum microsporidia. Microsporidia are eukaryotic unicellular organisms, obligate intracellular parasites related to fungi, and include more than 1700 species belonging to more than 220 genera [4]. Microsporidia species infecting the European rabbit are *Encephalitozoon cuniculi* [5], *Enterocytozoon bieneusi* [6], and *Encephalitozoon intestinalis* [7]; all are species with zoonotic potential. Spores, the infectious stage of microsporidia, are transmitted through the feces, urine, or respiratory excretes of infected animals or persons, being released into the environment where they can be ingested or inhaled [8,9]. To date, the vertical transmission of *E. cuniculi* has been described in rodents, rabbits, carnivores, horses, and non-human primates [10].

*Encephalitozoon cuniculi* is the microsporidia most frequently found in domestic and laboratory rabbits, with cases reported over the five continents [11]. Infection with *E. cuniculi* was first described by Wright and Craighead in 1919 in a rabbit suffering from motor paralysis. They observed microorganisms in histological samples of the cord, brain, kidney, spleen, and urine [12], later named *Encephalitozoon cuniculi* (syn. *Nosema cuniculi*) by Levaditi, Nicolau, and Schoen (1924) [5].

The European rabbit was first reported as an *E. bieneusi* host by del Águila et al. [6] in Spain. Since then, it has been detected in rabbits in Iran [13], China [14,15,16,17,18], and Egypt [7], particularly in farmed rabbits. The rarest species found in this mammal is *E. intestinalis*, first reported in rabbits in Egypt [7], and later in China [17] and Spain [19].

The diagnosis of microsporidia infection is confirmed by light [9] and electron microscopy [20,21], serology [9], immunohistochemistry, histology [22], or molecular analysis.

Polymerase chain reaction (PCR) provides a versatile tool for detecting microsporidia, along with differentiating species and genotypes in biological samples of infected patients or animals [9]. Four genotypes based on the number of 5′-GTTT-3′ repeats in the internal transcribed spacer (ITS) of the rRNA have been described in *E. cuniculi*: genotype I (three repeats), genotype II (two repeats), genotype III (four repeats), and genotype IV (five repeats) [23]. Genotypes I, II, and III have been found in several bird species, rodents, carnivores, artiodactyls, non-human primates, and humans worldwide [24,25], while genotype IV has been detected in cats, dogs, marmots, and immunosuppressed humans [26]. In rabbits specifically, genotype I is the most frequent genotype [27] found, while genotype II has been reported in domestic rabbits from Slovakia [28] and China [17], and genotype III only in domestic rabbits from Slovakia [28]. For *E. bieneusi*, more than 470 genotypes based on ITS sequence analysis have been identified in humans and animals worldwide. Genotypes with zoonotic potential have also been identified in rabbits in Spain [29] and China [14,15,16,17,18].

Regarding the clinical signs of infection in rabbits, *E. cuniculi* infection in some cases is asymptomatic, but the appearance of symptoms is common. The clinical signs of encephalitozoonosis may include neurologic manifestations, renal disorders, or ocular lesions [30], while available data suggest that the course of *E. bieneusi* and *E. intestinalis* infection in rabbits is asymptomatic [15,16,17,19,29].

Despite the fact that the European rabbit is considered the natural host of *E. cuniculi*, and the increase in reported *E. bieneusi* and *E. intestinalis* cases in this lagomorph, there is no data on microsporidia infection in rabbits from the Canary Islands (Spain). Therefore, the aim of this work was to analyze the prevalence and identify the species and genotypes of microsporidia in rabbits in Tenerife in the Canary Islands.

## 2. Materials and Methods

### 2.1. Ethical

Considering the work is based on fecal samples, no ethical approval was required for the described study. The fecal samples were donated by hunters that hunted wild rabbits during the legal hunting season or were collected by laboratory personnel on wild rabbit farms.

### 2.2. Study Area, Sample Collection, and Preparation

**Figure 1:**
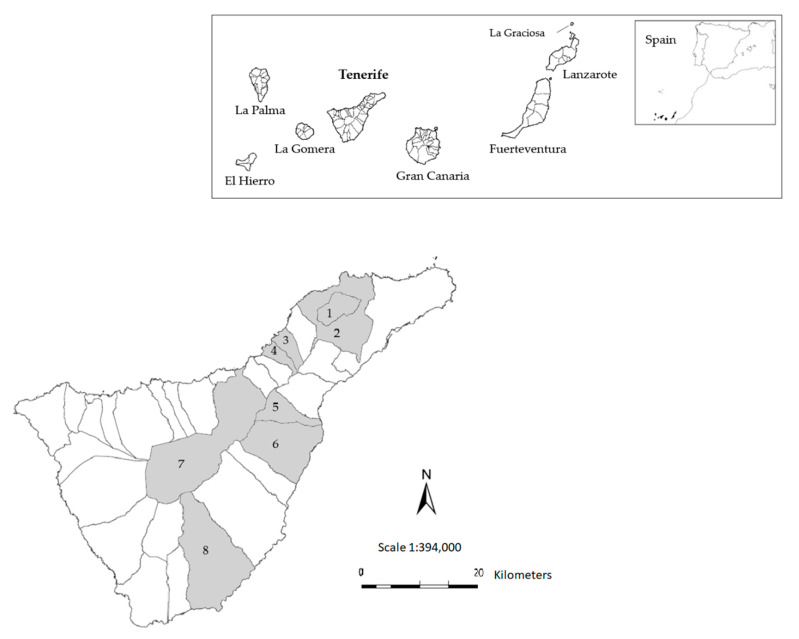
Map of the sampled locations on Tenerife island. The municipalities where fecal samples were collected are shown in gray—1: Tegueste, 2: San Cristóbal de La Laguna, 3: El Sauzal, 4: La Matanza de Acentejo, 5: Arafo, 6: Güímar, 7: La Orotava, 8: Granadilla de Abona. The original images were taken from Wikimedia Commons (https://commons.wikimedia.org/w/index.php?title=File:Mapa_Canarias_municipios.svg&oldid=478721455, accessed on 26 September 2022; https://commons.wikimedia.org/wiki/File:Islas_Canarias_(real_location)_in_Spain.svg, accessed on 26 September 2022) and Gobierno de Canarias (https://www3.gobiernodecanarias.org/medusa/mediateca/ecoescuela/?attachment_id=3333, accessed on 26 September 2022), in which the permission to copy, distribute, or adapt is established. Users: Júlio Reis (https://commons.wikimedia.org/wiki/User:Tintazul, accessed on 26 September 2022), TUBS (https://commons.wikimedia.org/wiki/User:TUBS, accessed on 26 September 2022), GRAFCAN (https://www.grafcan.es/, accessed on 26 September 2022), and IDE Canarias (http://www.idecanarias.es/, accessed on 26 September 2022) (Source: Gobierno de Canarias).

The study was conducted in eight municipalities of Tenerife, Canary Islands, between 2015 and 2017 (Figure 1). A total of 50 fecal samples from wild rabbits were collected. The origin of the samples was: donated by hunters (*n* = 18); samples from rabbits found dead (*n* = 5); fresh environmental fecal samples (*n* = 7); and samples from wild rabbits temporarily housed on an “industrial farm” (*n* = 11) and “family farms” (*n* = 9). The industrial farm was located in Granadilla de Abona and the two family farms were located in La Matanza de Acentejo and Tegueste.

The samples from farmed rabbits were collected from cages containing 1–3 rabbits per cage. For each sampled rabbit, information including gender, location, and health status was recorded when possible (Supplementary Material, Table S1).

After collection, the samples were placed into sterile plastic containers until delivery to the laboratory, and then deposited in vials containing 2.5% aqueous (*w/v*) potassium dichromate (K_2_Cr_2_O_7_) solution. The samples were stored at 4 °C until the analysis.

### 2.3. DNA Extraction

DNA from ~500 μL of each fecal sample was extracted using the commercial FastDNA^®^ Spin Kit for Soil (MP Biomedicals, Solon, OH, USA) following the manufacturer’s instructions, with the homogenizer FastPrep-24TM 5G (MP Biomedicals, Solon, OH, USA) used as the spore disruptor.

### 2.4. PCR Amplification

A nested PCR was performed in an XP Cycler (Bioer Technology, Hangzhou, China) using generic microsporidia primers described by Katzwinkel-Wladarsch et al. [31], amplifying the partial sequence of the 16S rRNA gene, the whole internal transcribed spacer region (ITS), and the partial sequence of the 5.8S rRNA gene.

The first PCR contained 0.15 μL of Taq DNA polymerase (5 U/ μL) (VWR), 0.1 μL of each primer (MSP1, MSP2A and MSP2B) (10 μM), 2.5 μL of dNTPs mix (200 μM) (Bioline, London, UK), 1.25 μL of MgCl_2_ (25 mM) (VWR), 2.5 μL of 10x key buffer (15 mM Mg^2+^) (VWR), and 1 μL of DNA template and water, to a total volume of 25 μL.

For the second PCR, the mixture was identical except that secondary primers (MSP3, MSP4A, and MSP4B) and 1 μL of primary PCR product were used. Each PCR reaction was then subjected to 35 cycles of denaturation at 94 °C for 45 s, annealing at 54 °C for 45 s, and extension at 72 °C for 1 min, with an initial denaturation at 94 °C for 3 min and a final extension step at 72 °C for 7 min [32].

PCR reactions were visualized on 1.5% (*w*/*v*) agarose gels (Fisher Bioreagents, Madrid, Spain) stained with REALSAFE Nucleic Acid Staining Solution (20,000 X, REAL, Durviz S.L., Valencia, Spain).

### 2.5. Sequencing and Sequencing Data Analysis

The nested PCR products with sizes ranging from 300 to 500 bp were sequenced at Macrogen Spain, with the secondary primers in both senses.

The sequences obtained using the Sanger method were interpreted with the MEGA X software [33], subsequently analyzed with the basic local alignment search tool (BLAST), and the identity confirmed by homology comparison.

## 3. Results

### 3.1. Prevalence of Microsporidia in Fecal Samples

Microsporidia DNA was detected in 7 out the 50 (14%) fecal samples, more specifically: 3 in La Orotava (30%; 3/10), 2 in Granadilla de Abona (14.3%; 2/14), 1 in San Cristóbal de La Laguna (33.3%; 1/3), and 1 in El Sauzal (12.5%; 1/8), while there were no positive results in La Matanza de Acentejo (0.0%; 0/5), Tegueste (0.0%; 0/5), Arafo (0.0%; 0/4), or Güímar (0.0%; 0/1).

A total of three positive samples were obtained from environmental fecal samples (42.9%; 3/7), two from single-caged rabbits on an industrial farm (18.2%; 2/11), one from hunted rabbits (5.6%; 1/18), and one from a rabbit found dead (20%; 1/5).

Among the sampled farms, on the industrial farm located in Granadilla de Abona, two positive results were obtained (18.2%; 2/11), but no positive samples were identified on the family farms located in La Matanza de Acentejo (0.0%; 0/5) or Tegueste (0.0%; 0/4).

### 3.2. Sequencing and Homology Comparison

Among the seven positive samples detected in rabbits, two were identified as *E. cuniculi* (28.6%; 2/7) and five as unknown microsporidia species (71.4%; 5/7) (Table 1).

*Encephalitozoon cuniculi* genotype I was identified in two fecal samples (MicC41 and MicC43). ITS sequencing analysis shows 100% and 99.65% homology with several *E. cuniculi* isolated (Acc. Numb: AB713183.1, AL391737.2, AJ005581.1, L13332.1). The origin of these sequences was two fecal samples from wild single-caged farmed rabbits on the industrial farm located in Granadilla de Abona (GenBank accession numbers OP555070 and OP555067).

The sequence obtained from sample Mic66 (GenBank accession number OP555068) from the dead rabbit in El Sauzal shows the highest homologies with several *Tubulinosema* species: 94.72% homology with the *Tubulinosema loxostegi* sequence (JQ906779.1); and 94.01% with *Tubulinosema hippodamiae* (KM883009.1), *Tubulinosema suzukii* (MN631017.1), and *Tubulinosema ratisbonensis* (AY695845.1).

The undetermined species detected from the environmental samples from La Orotava, MicC28, and MicC30 (GenBank accession numbers OP555064 and OP555065, respectively), are identical. Both sequences show 87.97% homology with the *Encephalitozoon hellem* isolate (OM731713.1) and 91.82% correlation with the *Encephalitozoon romaleae* isolate (FJ026013.1), with a query cover value of 50% and 42%, respectively. The sequence MicC60 (GenBank accession number OP555066), also from an environmental sample from La Orotava, shows homology with two *Encephalitozoon hellem* isolates, 88.49% (OM731713.1) and 90.48% (JF836368.1), with a query cover value of 48% and 44%, respectively.

The sequence MicC80 (GenBank accession number OP555069) from the hunted rabbit from San Cristóbal de La Laguna shares 88.36% homology with two *Bryonosema plumatellae* isolates (AF484690.1, AF484691.1) with a query cover value of 42%, and 89.47% correlation with *Schroedera airthreyi* (AJ749819.1) with a query cover value of 38%.

## 4. Discussion

In Spain, microsporidia infection has been detected in humans [34,35], animals [6,19,29,36], and wastewater [37,38]. Previous studies identified *E. cuniculi* by PCR in an AIDS patient (genotype III) [35], Crohn’s disease patients [39], water samples from Madrid (genotypes I and III) [38], the Iberian lynx (*Lynx pardinus*) in southern Spain [40], and captive chimpanzees (*Pan troglodytes*) in Madrid (genotype I) [41], demonstrating the presence of this species in mainland Spain. Furthermore, other studies reported cases in domestic rabbits in Spain using indirect immunofluorescence testing [36], histopathological analysis [42,43], or serological analysis [44,45,46,47,48,49,50]. However, the molecular characterization of *E. cuniculi* in rabbits from Spain has not been reported previously.

Studies on microsporidia infection in wild European rabbits are scarce. To our knowledge, microsporidia infection is confirmed in only six studies, four of which are based on serological assays, while the other two studies employ molecular techniques (Table 2).

Serological studies on the detection of antibodies against *E. cuniculi* in wild rabbits were carried out in several countries, with positive results in the UK (100%; 3/3) [51], France (3.9%; 8/204) [52], Slovakia (44.7%; 21/47) [53], and Australia (24.7%; 20/81) [54]. In contrast, no positive results were found in other studies carried out on wild rabbits in the UK (0.0%; 0/175), (0.0%; 0/27), (0.0%; 0/60) [55,56,57], Italy (0.0%; 0/100) [58], Australia (0.0%; 0/823), or New Zealand (0.0%; 0/57) [59].

Small subunit rRNA gene (SSU rDNA) sequence data for microsporidia infections in wild rabbits are limited. To date, one case of *E. bieneusi* infection in a fecal sample from a wild rabbit in Madrid [6], and three cases of *E. bieneusi* (0.8%; 3/383) and one of *E. intestinalis* (0.3%; 1/383) in kidney samples from wild rabbits in Andalusia, southern Spain [19] have been reported (Table 2).

In the case of domestic (farmed, pet, or laboratory) rabbits, *E. bieneusi* is the most prevalent infection found in fecal samples from pet rabbits (15.41%; 90/584), followed by *E. cuniculi* (5.8%; 34/584) and *E. intestinalis* (2.74%, 16/584) in China [17]. In smaller sample size studies, the observed prevalence ranges from 0.0% in Spain [6,29] to 100% in Switzerland [60] and Egypt [21] for *E. cuniculi*; 0.0% in Germany [61,62,63] to 30.8% in Egypt [7] for *E. bieneusi*; and 0.0% in Spain [6,29], Germany [61,63], and Italy [64] to 7.7% in Egypt [7] for *E. intestinalis* (Table 2). In other studies, also conducted in China, *E. bieneusi* is detected in the fecal specimens of farmed rabbits, with the prevalence in different studies of 0.94% (4/426) [15], 2.8% (9/321) [16], and 10.2% (22/215) [14]. In the latter studies, only *E. bieneusi*-specific primers are used (Table 2).

**Table 2:** Molecular studies of microsporidia in rabbits.

Wild rabbits					
Europe					
Country	Diagnostic Method	Prevalence (%) (Positive/Total)	Microsporidia (*n*)	Genotype (*n*)	Reference
Spain	PCR	14.3% (1/7) feces	*E. bieneusi* (1)	–	[6]
	PCR	0.8% (3/383) kidney 0.3% (1/383) kidney	*E. bieneusi* (3) *E. intestinalis* (1)	–	[19]
**Domestic rabbits**					
**Europe**					
Austria	PCR ^1^	0% (0/12) CSF 0% (0/32) urine 80% (4/5) lens	*E. cuniculi* (4)	–	[65]
France	Nested-PCR ^1^	80% (4/5) male * (brain, kidneys, liver) 80% (8/10) pregnant * (brain, kidney, lung) 56.5% (13/23) fetuses * (brain, kidney, lung, placenta)	*E. cuniculi*	I	[66] (P.J.R. Baneux pers. comm.; 2022)
Germany	Nested PCR (RFLP)	0% (0/3) feces	*–*	–	[62]
	Nested PCR (RFLP)	10.5% (2/19) CSF 39.5% (15/38) urine	*E. cuniculi* (16)	–	[61]
	Nested PCR (RFLP)	48.7% (18/37) urine	*E. cuniculi* (18)	–	[63]
	Real-time PCR ^1^	54.5% (30/55) at least one organ (brain, kidney, lungs, liver, heart, intestine)	*E. cuniculi*	–	[67]
	Real-time PCR ^1^	100% (3/3) CSF 36.7% (18/49) urine	*E. cuniculi*	–	[68]
Italy	PCR	36.4% (8/22) kidney	*E. cuniculi* (8)	–	[64]
Poland	Real-time PCR ^1^	26.21% (27/103) urine	*E. cuniculi* (27)	–	[69]
Slovakia	Culture PCR ^1^	Case report (brain, kidney, feces)	*E. cuniculi*	–	[70]
Spain	PCR	25% (3/12) feces	*E. bieneusi* (3)	–	[6]
	PCR	21% (4/19) feces	*E. bieneusi* (4)	D (1)	[29]
Switzerland	PCR–RFLP ^1^ WB	100% (9/9) in all samples (brain, kidney, urine)	*E. cuniculi* (9)	I	[60]
UK	PCR ^1^	33.3% (3/9) lens tissue	*E. cuniculi* (3)	–	[71] (R.F. Sanchez, pers. comm.; 2022)
**Asia**					
China	Nested PCR ^2^	10.2% (22/215) feces	*E. bieneusi* (22)	CHN-RD1 (12) D (3) Type IV (2) Peru6 (1) I (1) CHN-RR1 (1) CHN-RR2 (1) CHN-RR3 (1)	[14]
	Nested PCR ^2^	0.94% (4/426) feces	*E. bieneusi* (4)	D (4)	[15]
	Nested PCR ^2^	2.8% (9/321) feces	*E. bieneusi* (9)	J (5) BEB8 (3) Type IV (1)	[16]
	Nested PCR	15.4% (90/584) feces 5.8% (34/584) feces 2.7% (16/584) feces 0.9% (5/584) feces	*E. bieneusi* (90) *E. cuniculi* (34) *E. intestinalis* (16) Co-infection ** (5)	SC02 (39) I (21), N (13), J (6), CHY1 (1), SCR01 (1) SCR02 (1) SCR04 (1) SCR05 (2) SCR06 (2) SCR07 (3) I (19), II (15) SC02 + I (3) J + II (1) J + I (1)	[17]
	Nested PCR ^2^	Case report (feces)	*E. bieneusi*	I (2) Peru6 (4)	[18]
Iran	Nested PCR	10% (1/10) feces 10% (1/10) feces	*E. bieneusi* (1) *E. cuniculi* (1)	–	[13]
	Nested PCR ^1^	3.3% (2/60) brain	*E. cuniculi*	–	[72]
	Nested PCR ^1^	59.6% (34/57) brain	*E. cuniculi*	–	[72]
	Semi-nested PCR ^1^	32% (16/50) urine	*E. cuniculi* (16)	I (13)	[73]
Japan	Culture PCR ^1^	Case report (kidney)	*E. cuniculi*	I	[74,75]
	Nested PCR ^1^	7.78% (20/257) urine 0% (0/314) feces	*E. cuniculi*	I	[76]
	Nested PCR ^1^	33.3% (1/3) urine 5.6% (6/107) feces	*E. cuniculi*	I	[76]
Turkey	PCR ^1^	Case report (eye)	*E. cuniculi*	I	[77]
	PCR ^1^	Case report (brain)	*E. cuniculi*	I	[78]
	PCR ^1^	63% (19/30) eye 0% (0/25) blood 0% (0/24) kidney 0% (0/24) brain 0% (0/9) lung 0% (0/7) placenta 0% (0/2) liver 0% (0/2) heart	*E. cuniculi*	I	[79]
**America**					
Brazil	PCR	2.56% (11/429) feces	*Encephalitozoon* spp.	–	[80]
Canada	Real-time PCR ^1^	32.4% (11/34) urine	*E. cuniculi*	–	[81]
USA	PCR–RFLP ^1^	Case report (kidney)	*E. cuniculi*	Ia	[82]
	PCR ^1^ HMA	Case report (lens tissue)	*E. cuniculi*	I	[83]
	PCR ^1^	Case report (lens tissue)	*E. cuniculi*	Ia	[82]
	PCR ^1^	Case report (lens tissue)	*E. cuniculi*	–	[84]
**Oceania**					
Australia	PCR–RFLP ^1^	Case report (urine)	*E. cuniculi*	I	[5,85]
**Africa**					
Egypt	PCR ^1^	2.85% (1/35) urine	*E. cuniculi* (1)	–	[86]
	PCR	30.8% (4/13) feces 7.7% (1/13) feces	*E. bieneusi* (4) *E. intestinalis* (1)	–	[7]
	PCR ^1^	100% (150/150) in all samples (brain, eyeball, liver, kidney)	*E. cuniculi* (10)	–	[21]

^1^. *E. cuniculi*–specific primers; ^2^. *E. bieneusi*–specific primers. PCR–RFLP = restriction fragment length polymorphism; WB = Western blot; HMA = heteroduplex mobility shift analysis. * At least one organ; ** Co-infection with *E. bieneusi* and *E. cuniculi*.

The estimated prevalence of microsporidia infection depends on the diagnostic method employed, the type of sample, and the host habitat.

Direct methods, such as PCR, are suitable for the detection of microsporidia in an active infection with spore shedding, but could lead to underestimating the prevalence due to intermittent spore shedding periods, such as at the beginning of the primary infection or in chronic infections [9].

Considering the type of sample, the shedding of microsporidia spores in urine appears to be more common than in feces, as reported in the studies carried out by Valencakova et al. [28] and Kimura et al. [76]. The brain and kidney are the most frequently parasitized organ by *E. cuniculi* [66,67], followed by lung, heart, liver, and intestine. In this work, only fecal samples were analyzed, thus, the prevalence of *E. cuniculi* could be underestimated.

*Enterocytozoon bieneusi* and *E. intestinalis* are often detected in rabbit feces (Table 2), but the absence of these two species may be explained by the low prevalence observed in rabbit populations in Spain and the limited sampling available for analysis in this study (Table 2).

With respect to the host habitat, a higher prevalence has been observed in farmed rabbits, possibly due to the poor hygiene and overcrowding that is often found when rearing rabbits at commercial farms [15,87]. This is in agreement with the results obtained in this work, where *E. cuniculi* is only detected in wild rabbits kept temporarily on farms, with no positive results found in wild hunted rabbits or in environmental fecal samples. All sampled rabbits were wild-raised, but some of them were temporarily housed on industrial (*n* = 11) or family farms (*n* = 9) at the time of sampling, which could have been a risk factor for acquiring the *E. cuniculi* infection.

The origin of *E. cuniculi* infection in rabbits in Tenerife is unknown. Despite the limited sample size, the prevalence of *E. cuniculi* obtained in fecal samples in this study (4%; 2/50) is similar to that found in fecal samples from pet rabbits in China (5.8%; 34/584) [17] and from farmed rabbits in Japan (5.6%; 6/107) [76] using PCR.

A retrospective study carried out between 2000 and 2018 in northern Spain identified encephalitozoonosis, using histology, as the most frequent parasitic disorder found in domestic rabbits, while no cases were observed in wild rabbits [43]. With regard to previous data, the domestic (farmed and pet) rabbit population can be considered a reservoir of *E. cuniculi* infection in Spain, in contrast to the wild rabbit population.

Undetermined microsporidia species were detected in 5 out of the 50 (10%) rabbit samples from Tenerife. The primers used in this work were not specific and amplified the SSU rDNA of a wide range of microsporidia species. The novel microsporidian sequences detected in the rabbit feces may belong to microsporidia that pass through the digestive system with food or water, as was previously suggested for similar “orphan” sequences discovered in humans [88] and animals [29].

The sequence MicC66 (GenBank accession number OP555068) obtained from the dead rabbit shows the highest homologies (>94%) with several sequences belonging to the genus *Tubulinosema*, all isolated from insects: *T. loxostegi*, isolated from *Loxostege sticticalis* [89]; *T. hippodamiae*, isolated from *Hippodamia convergens* [90]; *T. suzukii*, isolated from *Drosophila suzukii* [91]; and *Tubulinosema ratisbonensis*, isolated from *Drosophila melanogaster* [92]. As the sequence shares less than 95% [93] correlation with *Tubulinosema* species, it may be a closely related non-*Tubulinosema* species with an insect source.

The sequence MicC80 (GenBank accession number OP555069) shares homology with sequences of *Bryonosema plumatellae* and *Schroedera airthreyi*, both isolated from a freshwater bryozoan of the genus *Plumatella* sp. [94,95]. The correlation of less than 85% and the low query cover value with the closest known species suggest that it is an undescribed genus or family of microsporidia [93].

Although the potential for the wild European rabbit to be a zoonotic source of microsporidia infection is relatively low [6,19], the domestic (farmed or pet) rabbit should be considered a source of human pathogenic microsporidia, especially for animal owners and farm keepers. *Encephalitozoon cuniculi* genotype I has been previously detected in several wild and farmed mammals and birds [24], as well as in immunocompromised [96] and immunocompetent humans [97]. Therefore, the transmission of this infection could pose a risk to public and veterinary health.

## 5. Conclusions

This study provides molecular data on microsporidia infection in rabbits in Tenerife, Canary Islands, Spain. The overall prevalence of microsporidia was 14.0%, with five cases of undetermined microsporidia species and two cases of *E. cuniculi*, all detected in fecal samples.

## Figures and Tables

**Table 1:** Microsporidia detected in rabbit fecal samples and location in Tenerife, Canary Islands (Spain).

Sample ID	Microsporidia	Location	Management
MicC28	Undetermined	La Orotava	Environmental
MicC30	Undetermined	La Orotava	Environmental
MicC41	*Encephalitozoon cuniculi*	Granadilla de Abona	Farm
MicC43	*Encephalitozoon cuniculi*	Granadilla de Abona	Farm
MicC60	Undetermined	La Orotava	Environmental
MicC66	Undetermined	El Sauzal	Hunted rabbit
MicC80	Undetermined	San Cristóbal de La Laguna	Found dead rabbit

## Data Availability

Not applicable.

## Supplementary Materials

The following supporting information can be downloaded at: https://www.mdpi.com/article/10.3390/biology11121796/s1, Table S1: Data of the rabbit samples analyzed in this study.
