# Supplementary material for: Molecular Detection of Microsporidia in Rabbits (Oryctolagus cuniculus) in Tenerife, Canary Islands, Spain
Source: Biology (Basel). 2022 Dec 10;11(12):1796. doi: 10.3390/biology11121796 (PMC9775083; doi:10.3390/biology11121796)
Supplement: Supplementary file 1 [file biology-11-01796-s001.zip › biology-1975348-supplementary.pdf]

Table S1. Data of the rabbit samples analyzed in this study.

Sample ID	Location	Management	Sex	Data
MicC1	Granadilla de Abona	Industrial farm	-	2015/10/20
MicC17	Granadilla de Abona	Industrial farm	-	2015/10/20
MicC21	Granadilla de Abona	Industrial farm	-	2015/10/20
MicC23	Granadilla de Abona	Industrial farm	-	2015/10/20
MicC27	La Orotava	Environmental	-	6月-17
MicC28	La Orotava	Environmental	-	6月-17
MicC29	La Orotava	Environmental	-	6月-17
MicC30	La Orotava	Environmental	-	6月-17
MicC32	Granadilla de Abona	Industrial farm	Male	2015/10/20
MicC36	Granadilla de Abona	Industrial farm	Male	2015/10/20
MicC40	Granadilla de Abona	Industrial farm	Female	2015/10/20
MicC41	Granadilla de Abona	Industrial farm	Female	2015/10/20
MicC43	Granadilla de Abona	Industrial farm	Female	2015/10/20
MicC44	Granadilla de Abona	Industrial farm	Female	2015/10/20
MicC46	Granadilla de Abona	Industrial farm	Female	2015/10/20
MicC48	Tegueste	Family farm	-	2015/10/20
MicC49	Tegueste	Family farm	-	2015/10/20
MicC51	Tegueste	Family farm	-	2015/10/20
MicC52	Tegueste	Family farm	-	2015/10/20
MicC54	Arafo	Hunted	Male	2015/10/20
MicC55	Arafo	Hunted	Female	2015/10/20
MicC57	Arafo	Hunted	Male	2015/10/20
MicC59	Arafo	Hunted	-	2015/10/20
MicC60	La Orotava	Environmental	Female	2015/10/26
MicC61	La Orotava	Environmental	-	2015/10/27
MicC62	La Orotava	Environmental	-	2015/10/27
MicC64	Güímar	Hunted	-	2015/11/4
MicC66	El Sauzal	Found dead	Female	2015/11/16
MicC68	Granadilla de Abona	Hunted	Male	2015/12/1
MicC69	Granadilla de Abona	Hunted	Female	2015/12/1
MicC70	Granadilla de Abona	Hunted	Female	2015/12/1
MicC71	El Sauzal	Hunted	-	2015/12/3
MicC72	El Sauzal	Hunted	Female	2015/12/3
MicC73	Tegueste	Found dead	-	2016/1/29
MicC75	El Sauzal	Hunted	-	2016/2/23
MicC76	El Sauzal	Hunted	-	2016/2/23
MicC77	El Sauzal	Hunted	-	2016/2/23
MicC78	San Cristóbal de La Laguna	Hunted	-	2016/3/24
MicC80	San Cristóbal de La Laguna	Hunted	Male	2016/3/24
MicC85	La Matanza de Acentejo	Family farm	-	2017/2/20
MicC86	La Matanza de Acentejo	Family farm	-	2017/2/20
MicC90	La Matanza de Acentejo	Family farm	-	2017/2/20
MicC92	La Matanza de Acentejo	Family farm	-	2017/2/20
MicC95	La Matanza de Acentejo	Family farm	-	2017/2/20
MicC100	El Sauzal	Found dead	-	2017/5/16
MicC101	El Sauzal	Found dead	Female	2017/5/15
MicC105	La Orotava	Hunted	Male	2017/5/25
MicC109	La Orotava	Hunted	Male	2017/6/21
MicC110	La Orotava	Hunted	Female	2017/6/21
MicC111	San Cristóbal de La Laguna	Found dead	-	2017/9/16
